# Prediction of $\beta$-Thalassemia carriers using complete blood count features

**DOI:** 10.1038/s41598-022-22011-8

**Published:** 2022-11-21

**Authors:** Furqan Rustam, Imran Ashraf, Shehbaz Jabbar, Kilian Tutusaus, Cristina Mazas, Alina Eugenia Pascual Barrera, Isabel de la Torre Diez

**Affiliations:** 1 Faculty of Computer Science and Information Technology, Khwaja Fareed University of Engineering and Information Technology, Rahim Yar Khan, Pakistan; 2 Information and Communication Engineering, Yeungnam University, Gyeongsan, 38541 Korea; 3 Sheikh Zayed Hospital and Medical College, Rahim Yar Khan, 64200 Pakistan; 4 Universidad Europea del Atlántico, Isabel Torres 21, 39011 Santander, Spain; 5 Universidad Internacional Iberoamericana, 24560 Campeche, Mexico; 6 Universidad Internacional Iberoamericana Arecibo, Puerto Rico, 00613 USA; 7 Universidade Internacional do Cuanza, Cuito, Bié Angola; 8 Fundación Universitaria Internacional de Colombia Bogotá, Bogotá, Colombia; 9 Department of Signal Theory and Communications and Telematic Engineering, University of Valladolid, Paseo de Belén 15, 47011 Valladolid, Spain

**Keywords:** Computational biology and bioinformatics, Health care

## Abstract

$\beta$-Thalassemia is one of the dangerous causes of the high mortality rate in the Mediterranean countries. Substantial resources are required to save a $\beta$-Thalassemia carriers’ life and early detection of thalassemia patients can help appropriate treatment to increase the carrier’s life expectancy. Being a genetic disease, it can not be prevented however the analysis of several indicators in parents’ blood can be used to detect disorders causing Thalassemia. Laboratory tests for Thalassemia are time-consuming and expensive like high-performance liquid chromatography, Complete Blood Count (CBC) with peripheral smear, genetic test, etc. Red blood indices from CBC can be used with machine learning models for the same task. Despite the available approaches for Thalassemia carriers from CBC data, gaps exist between the desired and achieved accuracy. Moreover, the data imbalance problem is studied well which makes the models less generalizable. This study proposes a highly accurate approach for $\beta$-Thalassemia detection using red blood indices from CBC augmented by supervised machine learning. In view of the fact that all the features do not carry predictive information regarding the target variable, this study employs a unified framework of two features selection techniques including Principal Component Analysis (PCA) and Singular Vector Decomposition (SVD). The data imbalance between $\beta$-Thalassemia carrier and non-carriers is handled by Synthetic Minority Oversampling Technique (SMOTE) and Adaptive Synthetic (ADASYN). Extensive experiments are performed using many state-of-the-art machine learning models and deep learning models. Experimental results indicate the superiority of the proposed approach over existing approaches with an accuracy score of 0.96.

## Introduction

Thalassemia is a hereditary genetic disorder that occurs due to mutations in the DeoxyriboNucleic Acid (DNA) of cells induced by insufficient production of Hemoglobin (Hb) in the body. Hb is a protein that allows Red Blood Cells (RBCs) to carry oxygen. The deficiency of Hb lowers the survival rate of RBCs resulting in a smaller number of RBCs flowing through the bloodstream leading to a limited supply of oxygen in the body which can be life-threatening. Two protein chains, $\alpha$, and $\beta$, are required to synthesize Hb. RBCs will not be able to carry oxygen efficiently if either of the aforementioned protein chains is insufficient. The $\alpha$-Thalassemia caused by less production of $\alpha$-protein chain, and $\beta$-Thalassemia caused by the absence or limited synthesis of $\beta$-protein chain, are the two forms of thalassemia disorder^1^. Symptoms of thalassemia range from mild to severe anemia which can cause organ damage and even death.

As of today, many countries are dealing with the growing rate of thalassemia, which has significantly increased disability and mortality worldwide. The $\beta$-Thalassemia is the most prevalent type of thalassemia which is common among the people of Mediterranean countries, hence also called ‘Mediterranean Anaemia’. Pakistan is one of the Mediterranean countries in which every year, approximately 5000–9000 children are diagnosed with $\beta$-Thalassemia disorder along with an estimated 5–7% carrier rate among the total populous^2^. According to the Thalassemia Federation of Pakistan, 25,000 children have been diagnosed with $\beta$-Thalassemia disorder, however, the actual figure is likely to be significantly higher, as many are living in areas where they do not have access to any thalassemia facility^3^. The Health Informatics (HI) integrates information technology to analyze and organize medical records efficiently. In recent years, the significance of HIs has increased due to the requirement for effective and secure management of medical records^4^. This resulted in an immense volume of medical data being analyzed using a variety of data mining techniques to acquire useful insights that can be utilized in the development of efficient systems to assist in the early diagnosis of genetic disorders like thalassemia.

Data mining has been extensively utilized in the medical field for the prognosis of available medical records. It involves the discovery of useful information from big data efficiently and cost-effectively. Data mining techniques are employed to process a large volume of raw data to discover useful knowledge. This procedure of uncovering novel patterns involves a series of steps, from data preprocessing to the prediction of future outcomes^5^. Therefore, data mining techniques can be effective in the development of a detection system that can help healthcare professionals in the prediction and early detection of $\beta$-Thalassemia. The carriers of $\beta$-Thalassemia do not show any symptoms of the disease and can be diagnosed by Complete Blood Count (CBC) test, high-performance liquid chromatography, or genetic test. CBC results contain several indicators that can be utilized to identify thalassemia carriers. Several approaches have been presented lately to detect thalassemia carriers^6–8^, however, such approaches are limited by the use of imbalanced datasets, lower classification accuracy, and less generalizability of models.

The current study proposes an approach to obtain high accuracy by resolving the data imbalance problem and increasing the efficacy of the feature selection approach. In summary, it makes the following contributions

- This study investigates the usefulness of data mining approaches in the accurate and robust screening of $\beta$-Thalassemia carriers and non-carriers based on several features from CBC.
- The dataset imbalance problem is resolved using two sampling approaches including Synthetic Minority Oversampling Technique (SMOTE) and Adaptive Synthetic (ADASYN).
- A unified framework of two feature reduction techniques including Principal Component Analysis (PCA) and Singular Value Decomposition (SVD) is proposed to acquire an optimum feature set for the training of classifiers.
- Extensive experiments are performed to evaluate the performance of the proposed approach using Decision Tree (DT), Gradient Boosting Machine (GBM), AdaBoost (ADA), Support Vector Classifier (SVC), Random Forest (RF), Extra Tree Classifier (ETC), and Logistic Regression (LR). In addition, deep learning models are also deployed including Long Short-Term Memory (LSTM), Gated Recurrent Unit (GRU), Convolutional Neural Network (CNN), and an ensemble called CNN-LSTM.
- Performance analysis is carried out with deep learning models and existing state-of-the-art approaches in terms of accuracy, precision, recall, and F1 score.

The rest of this paper is structured as follows. The following section covers the research papers related to the current study. “Materials and methods” are given in the third section. The fourth section provides “Experimental results” and in the end, the study is concluded.

## Related work

The manual approach for the diagnosis of $\beta$-Thalassemia carriers from patients’ data is time-consuming and costly. This urges for an expert predictive system that is capable of diagnosing $\beta$-Thalassemia carriers in less time and cost. Several pieces of research have proposed the use of machine learning and deep learning techniques to assist healthcare professionals to make informed decisions with less delay^9^. In this regard, we discuss the primary research in the literature which addresses the diagnosis of $\beta$-Thalassemia carriers using a variety of machine learning and deep learning models.

Sadiq et al.^8^ designed an aggregated classifier SGR-VC for the classification of $\beta$-Thalassemia carriers and non-carriers. The proposed classifier is an ensemble of SVC, GBM, and RF which is trained and evaluated on the CBC data of 5066 patients from Punjab Thalassaemia Prevention Programme, Pakistan (PTPP). The author opted for a simple CBC test of red blood cells to classify thalassemia carriers. The authors compared the performance of the proposed SGR-VC with SVC, GBM, and RF individually. Experimental results revealed that the suggested model with 93% accuracy was more efficient for the classification of $\beta$-Thalassemia carriers and non-carriers.

Egejuru et al.^10^ employed a manual questionnaire type model to estimate the danger of thalassemia in different age groups. Simple questions were selected and speeches and discussions were organized with related medical experts and the public. Multilayer Perceptron (MLP) was used to process the computational data. Results of the conferences were comparatively studied with actual lab results. Some environmental factors like living conditions, marital status, gender, death, and birth rate of 51 patients were also studied, in addition to medical variables like the size of spleen physiology and appearance of urine, diabatic grade, etc. Results show that 43% are patients with no disease, 31% are at high risk, 16% are at moderate risk and 11% are with the least risk factor.

Noferest et al.^11^ screened the iron-deficient anemic patients from $\beta$-Thalassemia minor by employing the data mining technique. The analysis was done by using the simple lab sampling of the CBC test. The CBC test was performed because it is cheap and consumes less time as compared to the other expensive and time taking tests. The data set was collected from Dr. Haidari’s laboratory situated in Zahedan city, Iran. The authors used several machine learning models like DT, naïve Bayes, bagging, SVC, and ADA for the experiments. The performance comparison of different models suggests that DT, naïve Bayes, ADA, SVC, and bagging obtained 96%, 76.6%, 80.2%, 95.5%, and 96.6% accuracy, respectively. The author concluded that the bagging classifier performed better.

Masala et al.^12^ designed a new model based on the radial basis function for the screening of normal persons from $\alpha$ and $\beta$-Thalassemia carriers. The dataset contains the records for 304 patients which is used with Probalistic Neural Network (PNN), k Nearest Neighbor (k-NN), and the Radial Basis Function (RBF). The classification is performed in two steps where in the first step, $\beta$-Thalassemia carriers are differentiated using the RBF and classified all the patients with 100% accuracy. In the second step, classification is performed by PNN and KNN with accuracy scores of 93% and 91%, respectively. Results showed that the RBF model was best due to its speed and efficiency. Barnhart-Magen et al.^12^ analyzed the patients suffering from thalassemia minor disease using a new screening method. The authors generated 1500 neural networks for the determination of sequences in the data sets. Every patient’s HB level, Mean Corpuscular Volume (MCV), Red cell Distribution Width (RDW), number of erythrocytes and platelets, and Mean Corpuscular Hemoglobin (MCH) tests were taken and analyzed for the screening. Experiments are performed using all three features separately. Results using three features show better performance.

Amendolia et al.^13^ conducted a study to classify Thalassemia patients using Pattern Recognition (PR) techniques. The author used two layer-based classifiers composed of SVM and k-NN and compare their results with the MLP classifier. The first layer of two layer-based classifier works to classify Thalassemia patients and healthy patients while the other layers classify the patients from the first layer into two types of thalassemia. The author only used the features that are relevant to the classification which include RCB, Ht, MCV, and HB without normalization. The results of this study showed that the MLP classifier performed better than the two-layer classifiers. Similarly^14^ used two methods for the diagnosis of thalassemia trait and normal ones. Through genetic programming, the author used a DT and neural network to classify thalassemia patients. Multiple regression analysis was used to check the values of the coefficients for the various DTs used in the classification. Results show a 90% accuracy using the MLP with two hidden layers.

**Table 1:** Summary of related work.

References	Overview	Models	Conclusion
^8^	Ensemble of best-performing machine learning classifiers under the majority voting criteria is proposed for a robust screening of $\beta$-Thalassemia carriers and non-carriers	SVM, GBM, and RF	Proposed SGR-VC yielded 93% accuracy on the test set
^10^	Investigated the risk of $\beta$-Thalassemia disorder in every age group by employing a manual questionnaire type model	MLP	Detection of $\beta$-Thalassemia disorder can be enhanced by incorporating Multilayer Perceptron (MLP)
^11^	Diagnosis of iron-deficient anemic patients from $\beta$-Thalassemia minor by utilizing a variety of data mining techniques	DT, NB, Bagging, SVM, and ADA	Bagging classifier performed efficiently in the diagnosis of iron-deficit patients
^12^	Screening of non-carriers from $\alpha -$Thalassemia and $\beta$-Thalassemia carriers using a bi-layered Radial Basis Function (RBF)	PNN, k-NN, and RBF	RBF performed well in the detection of non-carriers
^12^	Categorization of $\beta$-Thalassemia minor carriers by generating 1500 neural networks in the MATLAB. A two-fold study involving six features in the first phase and only three features in the second phase	Neural Networks	Proposed approach worked better with less and more significant features as compared to all features involved in experiments
^13^	Compared the performance of a two-layered machine learning model involving SVM and k-NN for first and second layer respectively, with MLP	MLP, SVM layered with k-NN	MLP performed comparatively better as compared to the two-layered learning model
^14^	Devised a machine learning model for the prognosis of thalassemia carriers and non-carriers	GP-based DT and MLP	MLP with two hidden layers carried out the prognosis with more efficacy than the GP-based DT
^15^	Screened a variety of groups of thalassemia disorder by integrating only significant features correlated with the thalassemia disorder such as MCV, and levels of hemoglobin	NB, DT, and MLP	NB and MLP showed better performance in the screening process

The authors use NB, DT, and MLP for the screening of various groups of thalassemia disorders in Ref.^15^. Data was collected by the characterization of CBC and the type and level of the hemoglobin. Out of various attributes of the CBC test, only MCV and levels of hemoglobin are selected for the analysis of the data. HPLC was used to analyze the different forms of hemoglobin. An accuracy of 94% is obtained using the NB while 92.5% with the MLP. A comparative analysis of discussed research works are provided in Table 1 which shows that lots of researchers have done work on $\beta$-Thalassemia predictions but still accuracy and efficiency a gap to work in this domain. The imbalanced dataset is a problem in base work^8^ that causes the model to over-fitting towards the majority class data and we work on this problem to achieve significant results.

**Figure 1:**
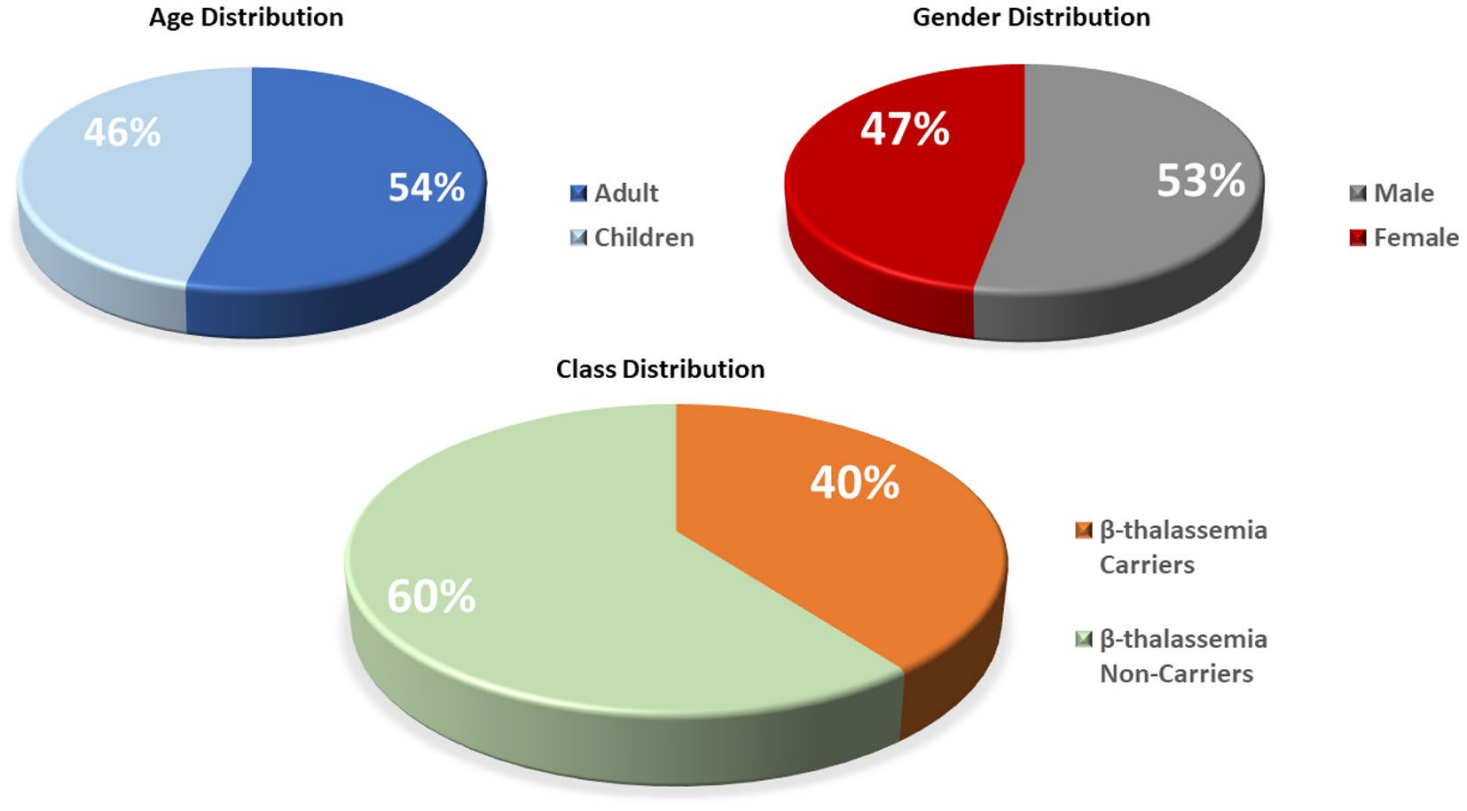
Dataset variable visualization.

## Materials and methods

This section discusses the dataset utilized in this study in addition to a detailed overview of the techniques employed for $\beta$-Thalassemia carriers classification using supervised machine learning. All methods were carried out in accordance with relevant guidelines and regulations.

### Dataset description

The dataset used in this research is collected from the database of PTPP^8^. PTPP is an institute of the Punjab Government of Pakistan that takes measures toward a Thalassemia-free country. The primary goal of PTPP is the cascade screening of $\beta$-Thalassemia carriers. In cascade screening whenever a patient with $\beta$-Thalassemia is diagnosed the complete screening of both parents’ families is performed. Given the fact that thalassemia is an inherited disorder, cascade screening encounters a considerable number of $\beta$-Thalassemia carriers. PTPP performs more than 300,000 tests every year. The records of 5066 individuals from 1000 families are incorporated in the current dataset. A total of 2015 individuals were diagnosed as $\beta$-Thalassemia carriers whereas 3051 were declared as $\beta$-Thalassemia non-carriers. Thalassemia carriers class indicates subjects who are diagnosed with $\beta$-Thalassemia major. On the other hand, $\beta$-Thalassemia non-carriers are healthy subjects. For the diagnosis, a complete blood count (CBC) is performed and Hb-Electrophoresis is carried out to confirm the carrier status of the individual. For a clear understanding of the dataset, we graphically present the dataset in terms of class, age, and gender-wise distribution in Fig. 1. The dataset involves a total of 12 features among which 9 features correspond to the attributes of CBC tests and 2 features contain demographic information regarding patients and 1 feature corresponds to the target label. These features are described in Table 2. In addition, the ratio of Thalassemia carriers regarding gender is 53% for males and 47% for females. Similarly, 54% of the carriers are adults while the rest 46% are children.

**Table 2:** Features and the relevant description.

Feature	Description	Data type
Age	Age of the patient	Numeric
Sex	Gender of the patient under-diagnosis	Categorical
RBC	Count of red blood cells in hemoglobin	Numeric
HB	The concentration of the hemoglobin protein molecules in the blood cells of a patient	Numeric
HCT	Hematocrit is the volume of RBCs in the blood	Numeric
MCV	Mean corpuscular volume measures the average size of RBCs	Numeric
MCH	Mean corpuscular haemoglobin measures the average volume of Hb in RBCs	Numeric
MCHC	Mean corpuscular haemoglobin concentration inside an individual RBC	Numeric
RDW	Distribution of width in RBCs	Numeric
PLT	The measure of the platelets in a volume of blood	Numeric
WBC	Count of white blood cells in a volume of blood	Numeric
Final finding	Diagnosis of the patient as $\beta$-thalassemia carrier or non-carrier	Categorical

### Proposed methodology

**Figure 2:**
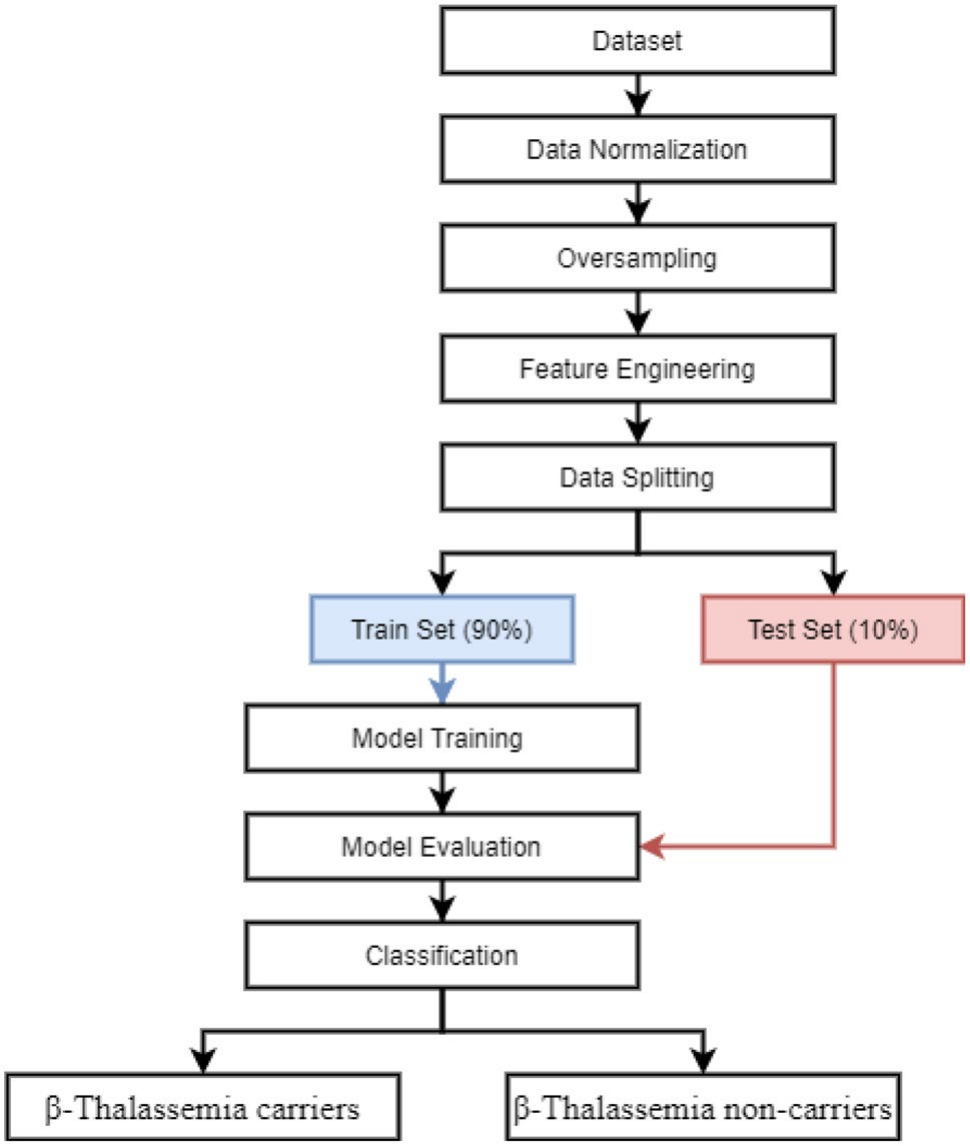
Flow of the proposed methodology.

This study utilizes the supervised machine learning approach for $\beta$-Thalassemia carriers classification. The flow of the proposed methodology is shown in Fig. 2.

First, we acquire the dataset from the study which is followed by the data normalization. Categorical data is normalized using ‘Label Encoder’ which is a technique for the transformation of categorical data into numeric data. Label encoder encodes the categorical values into numeric values with a 0 to N − 1 range. We implemented the encoding technique using the sci-kit-learn library LabelEnconder(). After that, we apply the data oversampling technique to balance the dataset for both target classes. Oversampling of data helps to increase the size of training data and also reduces the model over-fitting problem. We used SMOTE and ADASYN techniques for the oversampling. The number of samples after applying each resampling technique is shown in Table 3. Later feature engineering is performed to improve the performance of machine learning models. To make an appropriate feature set, this study proposes a hybrid approach where the features from PCA and SVD are combined. Data splitting is performed after feature engineering with a 0.9–0.1 ratio for training and testing, respectively. Models are trained using the training dataset and tested on the unseen test dataset. Performance is measured in terms of accuracy, precision, recall, and F1 score.

### Date resampling

The skewed distribution between target classes tends to produce ambiguous results because the learning model can only interpret the data samples from the majority class more effectively than that of the minority class. The authors in Ref.^16^ suggested the integration of resampling techniques to address the difficulty in the detection of the minority class label. In Ref.^17^ the authors reviewed that the poor performance of machine learning models is mainly due to the uneven distribution of class. The dataset under analysis is also subjected to the skewed distribution of target classes which results in a higher misclassification rate. To address this challenge we incorporated data resampling in this study. For this purpose, SMOTE and ADASYN are utilized to achieve a balanced distribution of target classes.

SMOTE is a statistical technique used to balance the dataset and solve the problem of over-fitting by adding new instances in the minority class. It randomly selects a single sample of data from the minority category and finds the nearest neighbors of that data sample. In SMOTE, the frequency is $k = 5$ for the selection of random data points and creating a new sample data for that line at the selected point.

ADASYN works analogously to SMOTE with trivial changes which involve the generation of samples that are more correlated with the ‘harder to learn’ samples. It selects the random point for the generation of minority class samples by finding linearly correlated values. It generates synthetic minority class samples which can be computed using

1 $$\begin{aligned} S_i = K_i + (K_u - K_i) x \lambda , \end{aligned}$$

where $\lambda$ is a random number: $\lambda ~ \varepsilon$ [0, 1] and $(K_u - K_i)$ is the vector difference in n-dimensional spaces. The number of samples after applying each resampling technique is shown in Table 3.

**Table 3:** Number of samples after data resampling.

Class	Original	SMOTE	ADASYN
$\beta$-Thalassemia carriers	2015	3051	3121
$\beta$-Thalassemia non-carriers	3051	3051	3051

### Data splitting

The estimation of the generalizability of a machine learning model is highly correlated with its performance on unseen data. Data splitting is performed to split the data into train and test sets. The train set is used by the machine learning models to learn and interpret the data instances following the target variable. Whereas, the test set is fed to the trained model to evaluate its efficacy. Owing to the small size of the dataset, we split the data into 0.9–0.1 ratios for the train and test set, respectively. The sample count after data splitting is shown in Table 4.

**Table 4:** Training and testing count after data splitting.

Class	Original		SMOTE		ADSYN	
	Training	Testing	Training	Testing	Training	Testing
$\beta$-Thalassemia carriers	1802	213	2746	305	2831	290
$\beta$-Thalassemia non carriers	2757	294	2745	306	2723	328
Total	5554	618	5491	611	4559	507

### Feature engineering

The dataset under consideration is comprised of features among which some contain high predictive information regarding the target variable whereas, some features do not contain or contain less predictive information. Therefore, reducing the number of input features facilitates an improvement in the model’s training. In this study, we utilized two well-known techniques including the PCA and SVD for the feature engineering of the dataset to optimize the performance of the classifiers.

**Figure 3:**
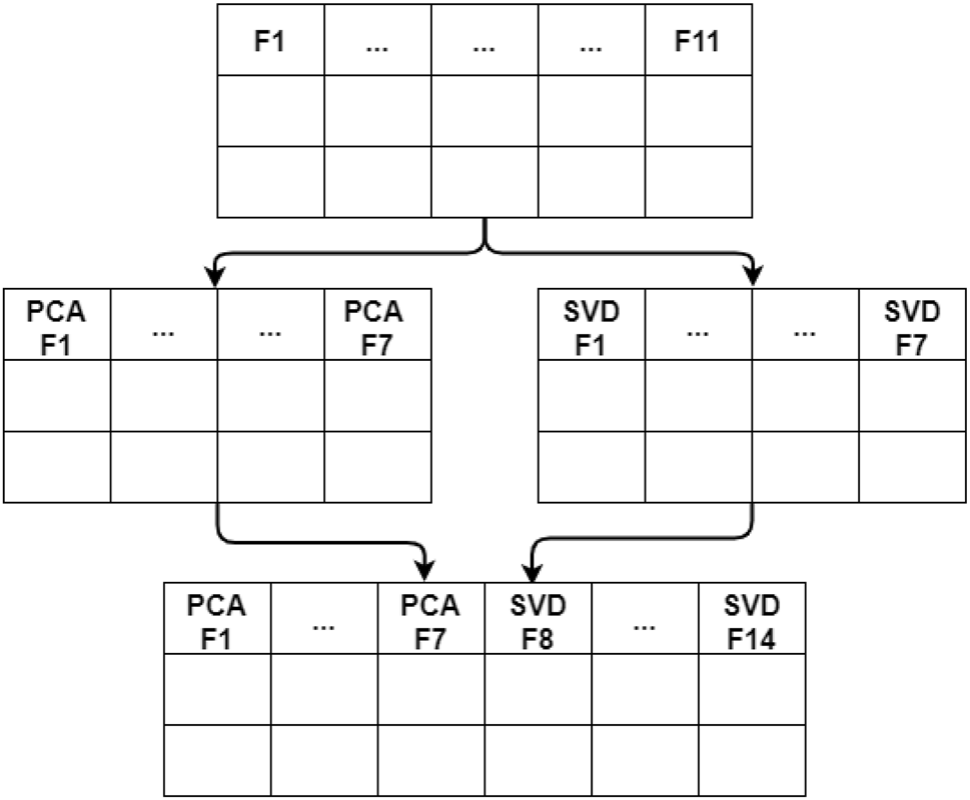
Visualization of combing features from PCA and SVD.

PCA is a quantitatively rigorous technique that projects high-dimensional data into a lower dimension without any loss of significant information. This technique is targeted at describing maximum variance with minimum reconstruction error by generating a new set of vector representations called principal components which are a linear combination of the original vector representations. PCA avoids redundancy as principal components tend to create an orthogonal space for the data. On the contrary, SVD is a generalized version of PCA as it infers the decomposition of a feature set of *n* features to a feature set of *k* features which allows the generation of a linear combination of the low number of linearly independent feature vectors which are easy to analyze and manipulate by the machine learning models. Whereas, the components with less significance are skipped using PCA.

In this study, we combined both feature reduction techniques’ results and make the training features set more significant. The dataset contains a total of 11 features and we reduced these 11 features into 9 using PCA and 9 using SVD. Then we combined these selected features into a single feature set of size 18 as shown in Fig. 3.

### Machine learning models

This study employs LR, GBM, DT, RF, ETC, SVC, and ADA to carry out classification tasks. Many hyperparameters are fine-tuned for machine learning models to optimize their performance.

#### Decision tree

DT has a tree-based structure in which the prediction made for each attribute is represented by an internal node, the prediction process is represented by each branch and the terminals or leaf nodes contain the target variable. DTs interpret the patterns from the train set based on the value of a single attribute set. This process repetitively takes place and is terminated when there is no further separation that can be made in the tree or when the output at the node is the same as the target variable^18^. DT is used with max_depth = 20 for this study.

#### Gradient boosting machine

GBM is an ensemble learning model which trains weak learners in a sequential, additive, and gradual manner. It integrates the loss function with the gradients which measures how well the coefficients of the model fit the underlying input data. GBM provides the benefit of cost function optimization by the user zhou2021developing^19^. GBM is used with max_depth = 300, learning_rate = 0.2, n_estimators = 350, and random_state = 52.

#### AdaBoost

ADA was initially developed to enhance the performance of a binary classifier by making use of iterations to gain information from the errors of the weak learners and then minimize the prediction error. It is an ensemble of weak learners which works by adjusting a weak learner on the input data and then fine-tuning more of the weak learners on the same input data concentrated on misclassified instances. This is done for the next weak learners to mainly target the wrongly predicted data instances^20^. ADA is used with n_estimators = 300, random_state=5, and learning_rate = 0.8.

#### Support vector classifier

SVC works by drawing the feature in N-dimensional space and then dividing the classes by drawing a hyperplane that separates the classes. Many hyperplanes are drawn the ideal hyper plan that divides the classes with the most distance from the features of other classes. The number of dimensions of a hyperplane is determined by the number of feature datasets contains. If the features of the dataset mapped on space are difficult to separate SVM uses a kernel function thus making SVC more flexible and effective. A kernel function maps the instance of data in higher dimensions which aids in separation by the hyperplane^21^. It is used with ‘poly’ kernel, C = 5.0, and a random_state of 500.

#### Random forest

RF is an ensemble technique that integrates unpruned trees developed by bootstrapping the samples of input training data and randomly selecting the features in the induction of trees. Every individual tree in RF forecasts a target variable. The target variable with a maximum number of votes is selected as the final prediction. Every decision tree is unique due to bootstrapping technique used by RF therefore the variance of RF decreases. RF can handle noise in datasets and performs exceptionally in classification^22^. For RF, 300 n_estimators are used with random_state = 5, while the max_depth is 03.

#### Extra tree classifier

ETC also called Extremely Randomized Trees Classifier is an ensemble classifier. Several DTs are constructed and for each tree whole training dataset is available rather than subsets of the dataset which is the case in RF. The trees are provided with a random sample of k features at each test node, from which the best features are selected by the DTs based on some mathematical parameters. Based on these random samples of features, a Multiple Decorrelated Decision Tree is constructed which produces the final output^23^. The hyperparameters for ETC are n_estimators = 200, random_state = 5, and the max_depth is 20.

#### Logistic regression

LR is a probabilistic model which classifies a given set of input X into a discrete set of output Y. It carries out classification tasks based on its output belonging to a target variable 0 or target value 1 which uses a sigmoid function to maximize the output within the given range of target variables^24^. The random_state is 1000, the solver is ‘liblinear’, multi_class = ‘ovr’, and C = 2.0 for experiments used in this study.

### Evaluation parameters

**Figure 4:**
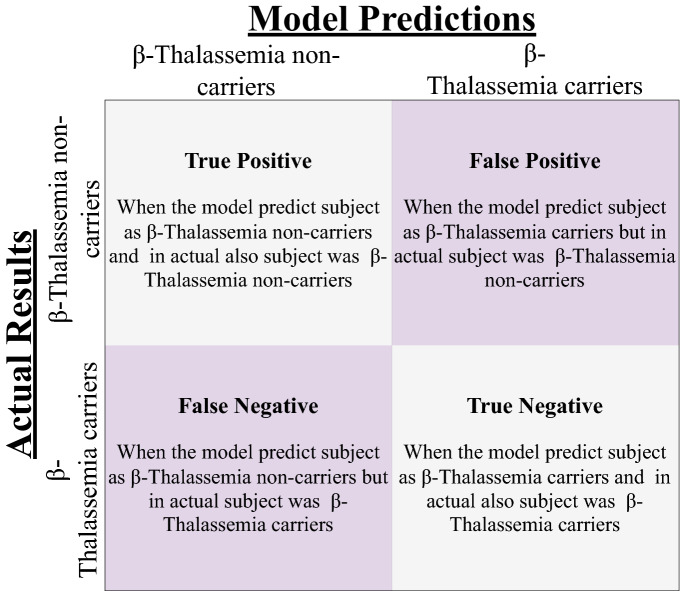
Definition of True Positive (TP) , True Negative (TN), False Positive (FP), False Negative (FN).

The evaluation methods assess the accuracy of models by analyzing test data and scoring them accordingly. In this study, the machine-learning models are evaluated by using four basic evaluation parameters including accuracy, precision, recall, and F1 score. We can calculate these evaluation parameters using confusion matrix values which are shown in Fig. 4.

We can define accuracy as:

2 $$\begin{aligned}&Accuracy= \frac{TP+TN}{TN+TP+FN+FP}, \end{aligned}$$

3 $$\begin{aligned}&\text {Positive Predictive Value (PPV)}= \frac{TP}{TP+FP}, \end{aligned}$$

4 $$\begin{aligned}&Sensitivity = \frac{TP}{TP+FN}, \end{aligned}$$

5 $$\begin{aligned}&F1-Score= 2*\frac{Precision * Recall}{Precision + Recall}, \end{aligned}$$

### Informed consent

Informed consent was obtained from all subjects and/or their legal guardian(s).

## Results and discussion

**Figure 5:**
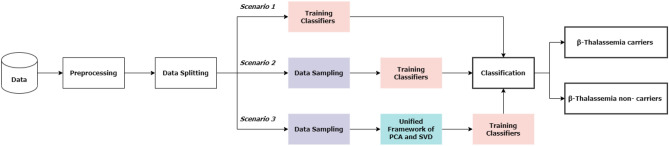
Details of three scenarios considered for experiments.

Extensive experiments are conducted in this study to evaluate the performance of classifiers in different scenarios. The first scenario follows the classification of $\beta$-Thalassemia carriers and non-carriers by training classifiers on the original dataset. The second scenario involves the classification of the target variables by machine learning models when trained with the resampled data. The third scenario employs resampling techniques along with a unified framework of two feature reduction techniques. These scenarios are illustrated in Fig. 5. This section presents the experimental results following the three aforementioned scenarios along with a detailed discussion. In addition to this, the performance of deep learning models following the three scenarios is also discussed in this section.

### Scenario 1: Classification results of ML models using original data

The original dataset corresponds to the skewed distribution of $\beta$-Thalassemia carriers and non-carriers with 12 features. In this scenario, the performance of the classifiers including ST, GBM, LR, RF, ETC, ADA, and SVC is evaluated on the original dataset. Table 5 shows the experimental results of ML classifiers utilized for the classification of $\beta$-Thalassemia carriers and non-carriers when integrated with original data. The results reveal that two tree-based models including ETC, RF, and a boosting classifier ADA achieve the highest accuracy score of 0.92 followed by similar weighted precision, recall, and F1 score. Whereas, SVC and LR with their ability to divide the target variables based on a decision boundary achieve a 0.91 accuracy score.

**Table 5:** Experimental results of ML classifiers using original data.

Classifier	Class	Accuracy	PPV	Sensitivity	F1 score
DT	$\beta$-Thalassaemia non-carrier	0.90	0.92	0.92	0.92
	$\beta$-Thalassaemia carrier		0.87	0.87	0.87
	Weighted average		0.90	0.90	0.90
GBM	$\beta$-Thalassaemia non-carrier	0.90	0.92	0.92	0.92
	$\beta$-Thalassaemia carrier		0.87	0.87	0.87
	Weighted average		0.90	0.90	0.90
ADA	$\beta$-Thalassaemia non-carrier	0.92	0.96	0.90	0.93
	$\beta$-Thalassaemia carrier		0.85	0.94	0.89
	Weighted average		0.92	0.92	0.92
SVC	$\beta$-Thalassaemia non-carrier	0.91	0.94	0.91	0.93
	$\beta$-Thalassaemia carrier		0.87	0.91	0.89
	Weighted average		0.91	0.91	0.91
RF	$\beta$-Thalassaemia non-carrier	0.92	0.94	0.93	0.93
	$\beta$-Thalassaemia carrier		0.88	0.90	0.89
	Weighted average		0.92	0.92	0.92
ETC	$\beta$-Thalassaemia non-carrier	0.92	0.93	0.93	0.93
	$\beta$-Thalassaemia carrier		0.89	0.89	0.89
	Weighted average		0.92	0.92	0.92
LR	$\beta$-Thalassaemia non-carrier	0.91	0.93	0.92	0.93
	$\beta$-Thalassaemia carrier		0.87	0.90	0.88
	Weighted average		0.91	0.91	0.91

**Figure 6:**
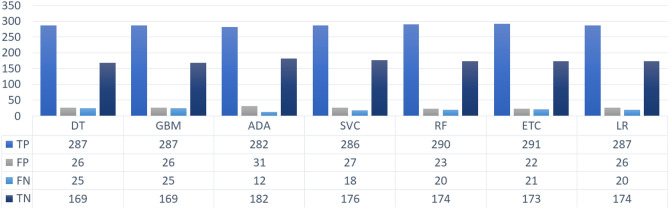
Count of correctly and incorrectly predicted instances of the test data.

Only accuracy, PPV, Sensitivity, and F1 score are not sufficient measures for the evaluation of a classifier in medical diagnosis. We also incorporated the count of correctly and incorrectly predicted instances to evaluate the performance of classifiers. Figure 6 reveals that the machine learning classifiers predicted the target variable with a high ratio of TP and TN, however, owing to the skewed distribution of target values, these high ratios of correctly predicted instances can be misleading^25^. Here, TP and FP refer to the count of correct and incorrect predictions $\beta$-Thalassemia non-carriers, respectively, TN and FN are the numbers of correct and incorrect predictions $\beta$-Thalassemia carriers respectively. The best performing tree-based models including RF, and ETC in this scenario show the highest count of correctly predicted instances whereas a significant difference can be observed between the TP and TN which is due to fewer samples of the minority class. On the other hand, ADA resulted in the lowest number of wrongly predicted $\beta$-Thalassemia carriers and the highest number of wrongly predicted $\beta$-Thalassemia non-carriers.

### Scenario 2: Classification results of ML models using oversampling techniques

This study intends to provide an accurate approach for the diagnosis of $\beta$-Thalassemia carriers and non-carriers. In the previous scenario, the data imbalanced problem resulted in inefficient training of the classifiers on the minority class i.e., $\beta$-Thalassemia carriers. This resulted in a higher ratio of wrongly predicted test samples from the target variable corresponding to $\beta$-Thalassemia carriers. To cope with the data imbalance problem, resampling techniques including SMOTE and ADASYN are implemented individually to acquire a balanced dataset for effective training of the machine learning models. The performance of ML classifiers corresponding to each oversampling technique is presented in this section.

#### Classification results of ML models using SMOTE

Table 6 presents the performance results of ML models when trained with data oversampled using SMOTE. It is indicated that the performance of classifiers is boosted when oversampling is integrated. Tree-based classifiers RF, and ETC yield the best performance with a 0.95 accuracy score followed by similar PPV, sensitivity, and F1 scores. These models are composed of an ensemble structure which results in better performance in the classification of $\beta$-Thalassemia carriers and non-carriers as compared to DT. The effectiveness of tree-based models over other models is because of their increasing number of trees which results in more generalization and reduction in variance^26^.

**Table 6:** Experimental results of ML classifiers with SMOTE.

Classifier	Class	Accuracy	PPV	Sensitivity	F1 score
DT	$\beta$-Thalassaemia non-carrier	0.92	0.93	0.92	0.93
	$\beta$-Thalassaemia carrier		0.92	0.92	0.92
	Weighted average		0.92	0.92	0.92
GBM	$\beta$-Thalassaemia non-carrier	0.92	0.92	0.92	0.92
	$\beta$-Thalassaemia carrier		0.91	0.91	0.91
	Weighted average		0.92	0.92	0.92
ADA	$\beta$-Thalassaemia non-carrier	0.94	0.96	0.93	0.95
	$\beta$-Thalassaemia carrier		0.92	0.96	0.94
	Weighted average		0.94	0.95	0.94
SVC	$\beta$-Thalassaemia non-carrier	0.92	0.96	0.89	0.92
	$\beta$-Thalassaemia carrier		0.89	0.96	0.92
	Weighted average		0.92	0.92	0.92
RF	$\beta$-Thalassaemia non-carrier	0.95	0.97	0.94	0.96
	$\beta$-Thalassaemia carrier		0.93	0.97	0.95
	Weighted average		0.95	0.95	0.95
ETC	$\beta$-Thalassaemia non-carrier	0.95	0.97	0.93	0.95
	$\beta$-Thalassaemia carrier		0.93	0.97	0.95
	Weighted average		0.95	0.95	0.95
LR	$\beta$-Thalassaemia non-carrier	0.93	0.96	0.91	0.93
	$\beta$-Thalassaemia carrier		0.90	0.96	0.93
	Weighted average		0.93	0.93	0.93

**Figure 7:**
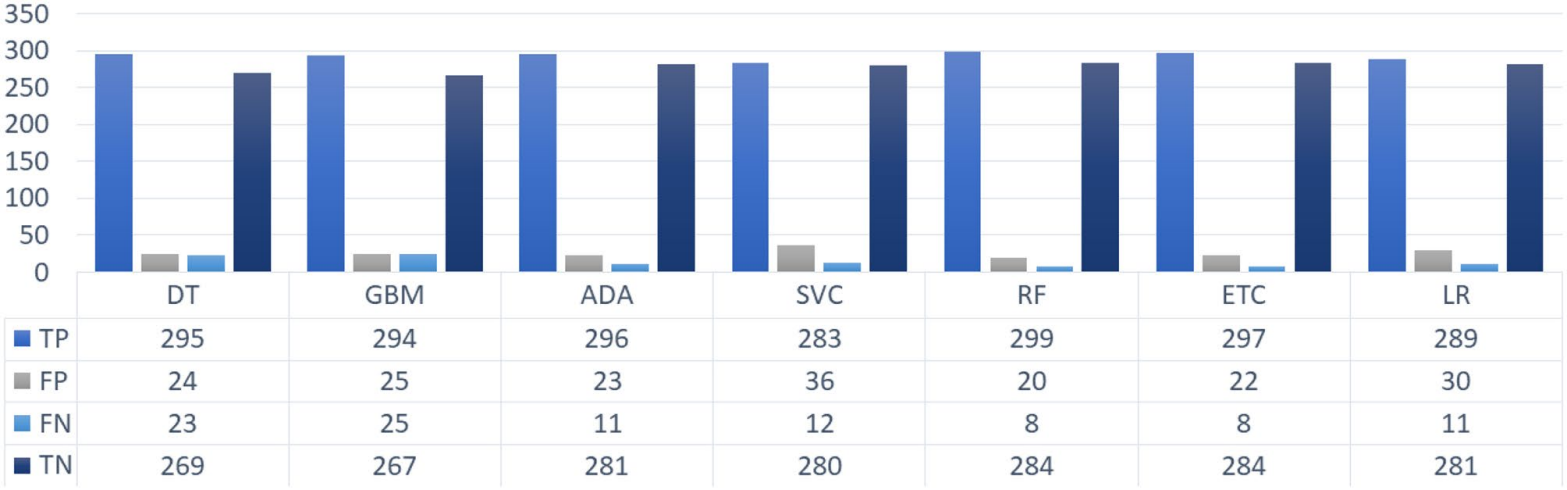
Count of correctly and incorrectly predicted instances by ML classifiers when trained SMOTE oversampled data.

For a better understanding of the performance of classifiers, we present the correctly and incorrectly classified instances using SMOTE in Fig. 7. A significant increase in the correctly classified $\beta$-Thalassemia carrier instances can be observed. RF has the lowest number of wrongly classified $\beta$-Thalassemia carriers and non-carriers and outperforms other models. Whereas, LR and SVC perform poorly with the highest number of wrongly classified instances which is a clear indication that these models are not suitable for the classification of the current dataset when subjected to oversampling with SMOTE.

#### Classification results of ML models using ADASYN

This study also considers using ADASYN for oversampling. Then, the same ML models discussed above are employed to carry out classification tasks. The primary difference between SMOTE and ADASYN is that the latter utilizes density distribution to generate minority samples and the former generates the same number of synthetic samples for the minority class^27^. Therefore, SMOTE generated 3051 samples for the minority class and ADASYN generated 3121 samples for the minority class. Table 7 shows that the performance of ML classifiers using ADASYN oversampled data is somewhat lower than that of SMOTE. However, RF and ETC remained the highest performing classifiers with a 0.94 accuracy score. In terms of other evaluation parameters such as PPV, sensitivity, and F1 score the aforementioned tree-based models also remained in the first place. Whereas GBM and SVC showed comparatively poor performance.

**Table 7:** Experimental results of ML classifiers with ADASYN.

Classifier	Class	Accuracy	PPV	Sensitivity	F1 score
DT	$\beta$-Thalassaemia non-carrier	0.92	0.95	0.87	0.91
	$\beta$-Thalassaemia carrier		0.88	0.96	0.92
	Weighted average		0.92	0.92	0.92
GBM	$\beta$-Thalassaemia non-carrier	0.90	0.88	0.93	0.90
	$\beta$-Thalassaemia carrier		0.92	0.88	0.90
	Weighted average		0.90	0.90	0.90
ADA	$\beta$-Thalassaemia non-carrier	0.91	0.94	0.89	0.91
	$\beta$-Thalassaemia carrier		0.89	0.94	0.91
	Weighted average		0.91	0.91	0.91
SVC	$\beta$-Thalassaemia non-carrier	0.90	0.96	0.84	0.90
	$\beta$-Thalassaemia carrier		0.85	0.97	0.91
	Weighted average		0.91	0.90	0.90
RF	$\beta$-Thalassaemia non-carrier	0.94	0.97	0.91	0.94
	$\beta$-Thalassaemia carrier		0.91	0.97	0.94
	Weighted average		0.94	0.94	0.94
ETC	$\beta$-Thalassaemia non-carrier	0.94	0.98	0.91	0.94
	$\beta$-Thalassaemia carrier		0.91	0.98	0.95
	Weighted average		0.95	0.94	0.94
LR	$\beta$-Thalassaemia non-carrier	0.91	0.93	0.88	0.91
	$\beta$-Thalassaemia carrier		0.89	0.93	0.91
	Weighted average		0.91	0.91	0.91

**Figure 8:**
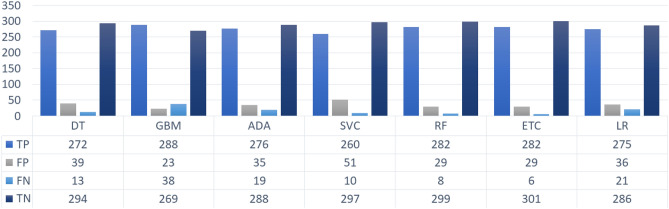
Count of correctly and incorrectly predicted instances by ML classifiers when trained on data oversampled using ADASYN.

The test sample involves a total of 618 instances among which 311 corresponds to the class of $\beta$-Thalassaemia non-carriers and 307 are the test instances of $\beta$-Thalassaemia carriers from which the highest ratio of incorrectly predicted samples is achieved by SVC as shown in Fig. 8. However, ETC and RF being the highest accurate models yield the lowest ratio of incorrect predictions. Overall, it can be observed that the tree-based models perform comparatively better whereas, the boosting model GBM shows poor performance.

### Scenario 3: Classification results of ML models using oversampling techniques integrated with unified framework of PCA and SVD

The dataset under analysis consists of twelve features among which all features do not carry predictive information regarding the target variable. This study intends to improve the performance of classifiers for the diagnosis of $\beta$-Thalassaemia carriers, therefore, we employed feature selection for effective training of the classifiers with only significant features. For this purpose, we proposed a unified framework of PCA and SVD which first chooses the nine most significant features with PCA and SVD individually and then combines these selected features into a single optimal feature set. Furthermore, the classifiers are trained with the combined feature set.

**Table 8:** Experimental results of ML classifiers using SMOTE integrated with unified framework of PCA and SVD.

Classifier	Class	Accuracy	PPV	Sensitivity	F1-Score
DT	$\beta$-Thalassaemia non-carrier	0.91	0.90	0.92	0.91
	$\beta$-Thalassaemia carrier		0.92	0.90	0.91
	Weighted average		0.91	0.91	0.91
GBM	$\beta$-Thalassaemia non-carrier	0.91	0.89	0.94	0.91
	$\beta$-Thalassaemia carrier		0.94	0.88	0.91
	Weighted average		0.91	0.91	0.91
ADA	$\beta$-Thalassaemia non-carrier	0.94	0.93	0.95	0.94
	$\beta$-Thalassaemia carrier		0.95	0.93	0.94
	Weighted average		0.94	0.94	0.94
SVC	$\beta$-Thalassaemia non-carrier	0.94	0.94	0.94	0.94
	$\beta$-Thalassaemia carrier		0.94	0.95	0.94
	Weighted average		0.94	0.94	0.94
RF	$\beta$-Thalassaemia non-carrier	0.96	0.96	0.95	0.96
	$\beta$-Thalassaemia carrier		0.95	0.96	0.96
	Weighted average		0.96	0.96	0.96
ETC	$\beta$-Thalassaemia non-carrier	0.96	0.96	0.95	0.96
	$\beta$-Thalassaemia carrier		0.96	0.96	0.96
	Weighted average		0.96	0.96	0.96
LR	$\beta$-Thalassaemia non-carrier	0.92	0.91	0.93	0.92
	$\beta$-Thalassaemia carrier		0.93	0.92	0.92

#### Classification results of ML models using SMOTE integrated with unified framework of PCA and SVD

Feature selection and expansion of feature set size boosted the performance of ML classifiers as shown in Table 8. The performance results of ML classifiers when trained on the oversampled dataset by SMOTE integrated with the unified framework of PCA and SVD reveal that the ensemble tree-based models such as ETC and RF show the best performance with a 0.96 accuracy score. Whereas, the remainder of the ML models including LR, DT, and GBM achieve 0.92, 0.91, and 0.91 accuracy scores, respectively which is comparatively lower. As for ADA and SVC, the increase in the number of features enhanced the predictive capability of these models as they achieved a 0.94 accuracy score.

**Figure 9:**
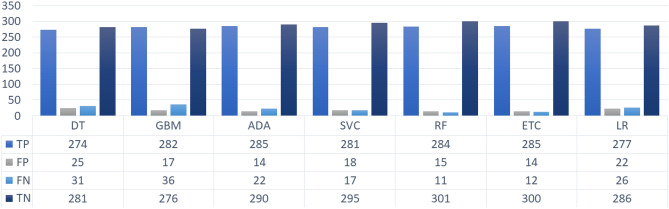
Count of correctly and incorrectly predicted instances by ML classifiers using SMOTE integrated with unified framework of PCA and SVD.

Figure 9 reveals that overall, the feature selection and expansion combined with SMOTE oversampled data increases the performance of the ML classifiers. PCA and SVD generate a feature set of attributes that are highly correlated with the target variable. Their unified framework results in a larger feature set comprised of significant features which improve the training of the classifiers hence boosting their performance. It is indicated that the number of correctly classified target variables has increased with the proposed approach. This shows the effectiveness of the proposed approach of combining oversampling technique with the unified framework of PCA and SVD.

#### Classification results of ML models using ADASYN integrated with unified framework of PCA and SVD

Table 9 reveals that the classifiers performed comparatively lower when integrated with feature union and ADASYN oversampling technique than with SMOTE. However, ETC showed better performance with a 0.96 accuracy score and 1.00 precision in the classification of the $\beta$-Thalassaemia non-carrier class. ADASYN oversamples the minority class following the learning difficulty of the minority class. More synthetic samples will be generated for the target class which is relatively harder to learn and interpret. Although ADASYN has been advocated to solve the problems faced by SMOTE, in literature it can be viewed that SMOTE outperforms ADASYN for classification tasks^28–30^ which is the case in this study. Overall performance of the classifiers is observed to decrease with ADASYN. The lowest accuracy score of 0.89 is achieved by GBM when subjected to training samples oversampled by ADASYN and the feature set generated by a unified framework of PCA and SVD.

**Table 9:** Experimental results of ML classifiers using ADASYN integrated with unified framework of PCA and SVD.

Classifier	Class	Accuracy	PPV	Sensitivity	F1 score
DT	$\beta$-Thalassaemia non-carrier	0.90	0.90	0.90	0.90
	$\beta$-Thalassaemia carrier		0.90	0.90	0.90
	Weighted average		0.90	0.90	0.90
GBM	$\beta$-Thalassaemia non-carrier	0.89	0.87	0.92	0.89
	$\beta$-Thalassaemia carrier		0.91	0.87	0.89
	Weighted average		0.89	0.89	0.89
ADA	$\beta$-Thalassaemia non-carrier	0.92	0.94	0.91	0.92
	$\beta$-Thalassaemia carrier		0.91	0.94	0.93
	Weighted average		0.92	0.92	0.92
SVC	$\beta$-Thalassaemia non-carrier	0.93	0.97	0.89	0.93
	$\beta$-Thalassaemia carrier		0.90	0.97	0.93
	Weighted average		0.93	0.93	0.93
RF	$\beta$-Thalassaemia non-carrier	0.95	0.99	0.91	0.95
	$\beta$-Thalassaemia carrier		0.92	0.99	0.95
	Weighted average		0.95	0.95	0.95
ETC	$\beta$-Thalassaemia non-carrier	0.96	1.00	0.92	0.96
	$\beta$-Thalassaemia carrier		0.93	1.00	0.96
	Weighted average		0.96	0.96	0.96
LR	$\beta$-Thalassaemia non-carrier	0.90	0.90	0.89	0.90
	$\beta$-Thalassaemia carrier		0.89	0.90	0.90
	Weighted average		0.90	0.90	0.90

**Figure 10:**
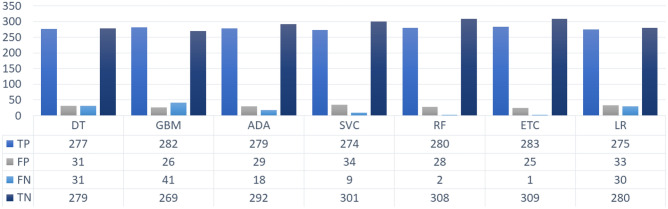
Count of correctly and incorrectly predicted instances by ML classifiers using ADASYN integrated with unified framework of PCA and SVD.

For a detailed evaluation of the performance of the proposed approach when integrated with ADASYN we present the count of correct and incorrect classified test instances in Fig. 10. ETC with the highest accuracy score classified the highest number of correct instances and the lowest ratio of incorrectly predicted test samples. Whereas, the remainder of the models show poor performance with 10.8%, 10.0%, 7.0%, 6.9%, 4.8%, 4.2%, and 10.1% wrong predictions by GBM, DT, ADA, SVC, RF, ETC, and LR, respectively.

**Figure 11:**
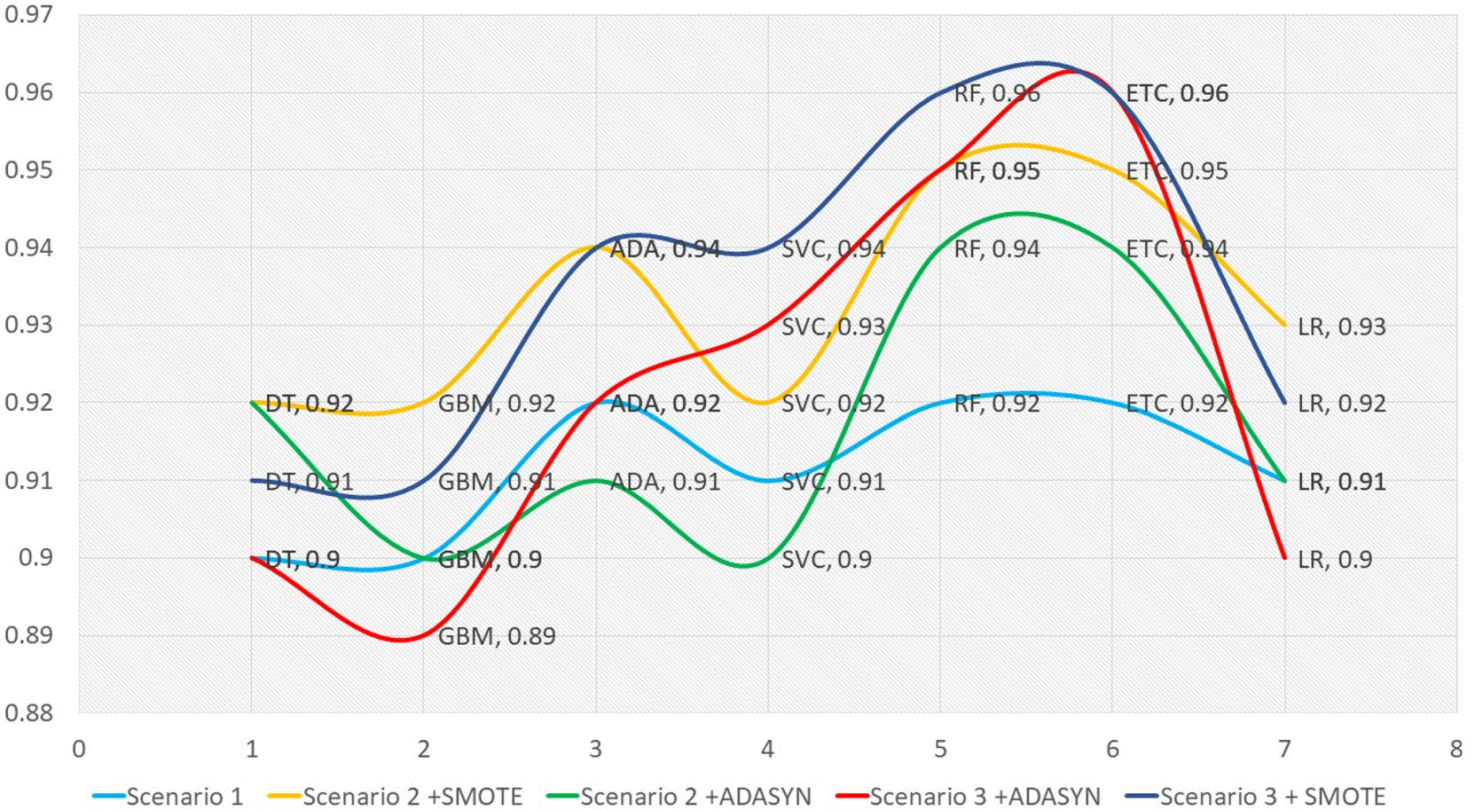
Comparative analysis of ML models in Scenario 1, Scenario 2, and Scenario 3.

The current study focuses on providing an accurate diagnosis of the $\beta$-Thalassaemia carrier by proposing a combined framework of oversampling technique and feature selection techniques. For this purpose, three scenarios are integrated into this study. The performance of ML classifiers corresponding to each scenario is discussed above. To see the overall performance of the proposed approach and other scenarios we have graphically presented the accuracy of each ML classifier in Fig. 11 which reveals that overall, the performance of the classifiers integrated into the proposed scenario 3 which involves oversampling with SMOTE and unified framework of PCA and SVD, is better as compared to other scenarios which show the efficacy of the proposed approach in diagnosing the $\beta$-Thalassaemia carriers and non-carriers. In terms of classifiers, the tree-based models involving an aggregated ensemble of DTs outperformed other ML classifiers. Whereas, in terms of oversampling technique, a significant improvement in the performance of classifiers can be observed as compared to the original data. Therefore, our proposed approach involving SMOTE as an oversampling technique and a unified framework of PCA and SVD as a feature selection and expansion technique stands out in diagnosing the $\beta$-Thalassaemia carriers using the dataset under consideration.

### Experimental results of deep learning networks

**Figure 12:**
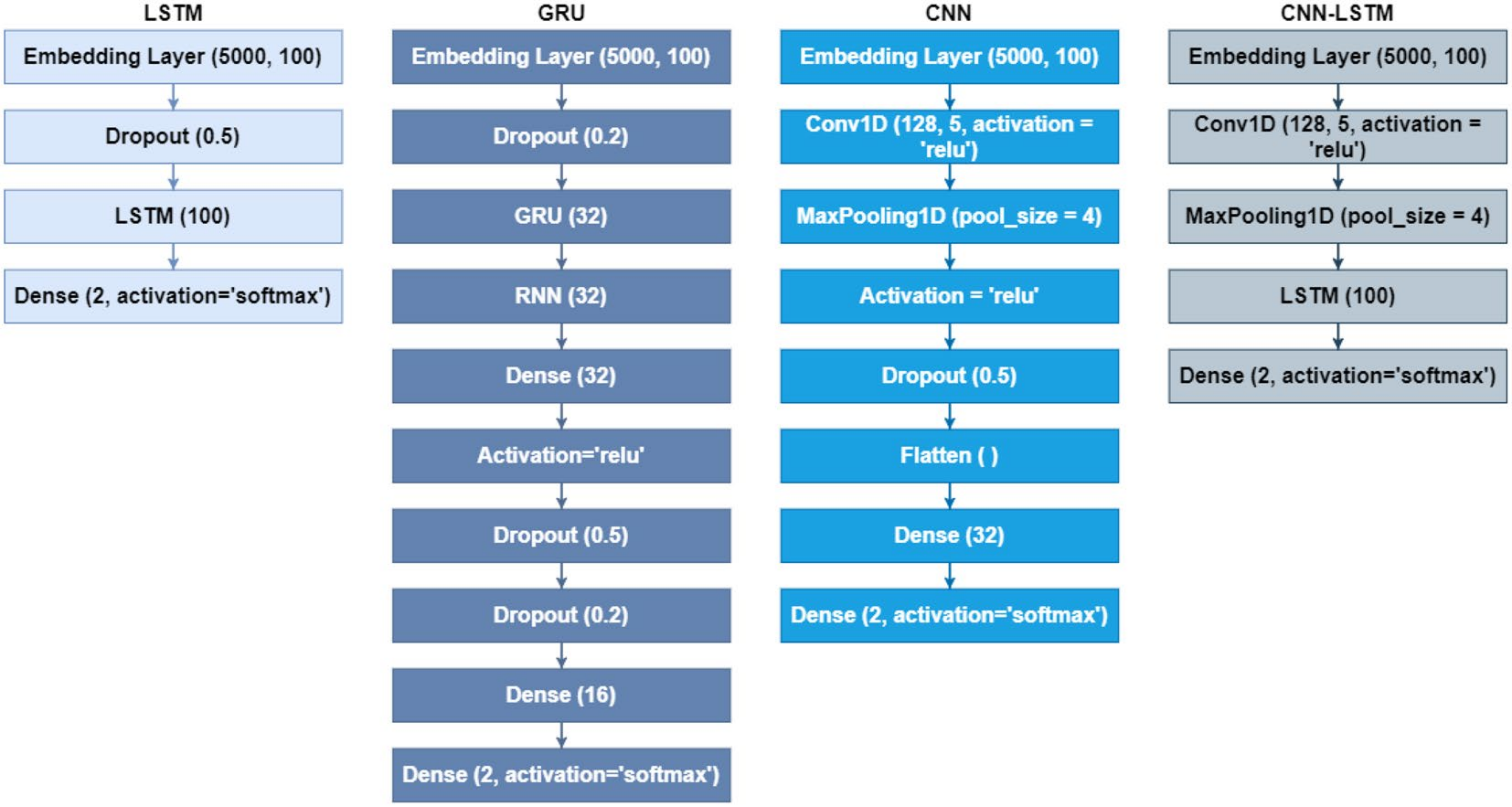
Count of correctly and incorrectly predicted instances of the test data.

This section presents the performance results of the neural networks integrated into the three aforementioned scenarios. Four neural networks including LSTM^31^, GRU^32^, CNN^33^, and CNN-LSTM^34^ are utilized in this study. Figure 12 illustrates the experimental settings of the aforementioned neural networks. Table 10 reveals that neural networks do not perform well regarding the classification task of $\beta$-Thalassaemia carriers and non-carriers. Neural networks provide high-quality results when the data under analysis is comprised of a large number of records. The above-mentioned neural networks are not able to efficiently carry out the classification task under consideration. This is mainly due to the small number of training samples fed into the input layers of networks for the models to interpret the hidden patterns. This shows that the proposed approach works efficiently with machine learning models.

**Table 10:** Experimental results of neural networks.

Scenarios	Classifiers	Accuracy	PPV	Sensitivity	F1 score
Scenario 1	LSTM	0.87	0.87	0.87	0.87
	GRU	0.89	0.89	0.89	0.89
	CNN	0.90	0.91	0.90	0.90
	CNN-LSTM	0.90	0.90	0.90	0.90
Scenario 2 (SMOTE)	LSTM	0.89	0.89	0.89	0.89
	GRU	0.90	0.90	0.90	0.90
	CNN	0.91	0.91	0.91	0.91
	CNN-LSTM	0.91	0.91	0.91	0.91
Scenario 2 (ADASYN)	LSTM	0.89	0.89	0.89	0.89
	GRU	0.87	0.87	0.87	0.87
	CNN	0.86	0.86	0.86	0.86
	CNN-LSTM	0.87	0.87	0.87	0.87
Scenario 3 (SMOTE)	LSTM	0.91	0.91	0.91	0.91
	GRU	0.91	0.91	0.91	0.91
	CNN	0.90	0.90	0.90	0.90
	CNN-LSTM	0.90	0.90	0.90	0.90
Scenario 3 (ADASYN)	LSTM	0.88	0.88	0.88	0.88
	GRU	0.90	0.90	0.90	0.90
	CNN	0.89	0.89	0.89	0.89
	CNN-LSTM	0.91	0.91	0.91	0.91

### Performance comparison of proposed study with previous approaches

The effectiveness of the proposed approach is investigated by comparing its performance with the previous state-of-the-art study performed to diagnose the $\beta$-Thalassaemia carriers using CBC tests of 5066 patients among which the $\beta$-Thalassaemia carrier target class comprise only 39.7% of the dataset which was collected from the database of PTPP^8^. The study utilized an ensemble of three statistical machine learning models including SVC, GBM, and RF, and achieved a 93% accuracy score. $\beta$-Thalassemia carriers are classified with 89% precision, 89% recall, and 90% F1 score, whereas, $\beta$-Thalassaemia non-carriers are predicted with 96% PPV, 93% sensitivity, and 93% F1 score. The variance precision of predicted target classes in the previous study is due to the skewed distribution of classes indicating that the classifiers are subjected to bias. Therefore, the F1 score for the diagnosis of $\beta$-Thalassaemia carriers is low as compared to that of $\beta$-Thalassaemia non-carriers. Table 11 reveals the superior performance of the proposed approach on a similar dataset when subjected to oversampling using SMOTE with ETC, RF, and ADASYN with ETC, and feature selection and expansion using the unified framework of PCA and SVD. It is also evident from this comparison, that the proposed approach with ETC or RF produces state-of-the-art results with low computation cost and less time consumption. Whereas, with the ensemble structure of three weak learners, the computation cost along with diagnosis time increases. This also shows the robustness of the approach proposed in the current study.

**Table 11:** Performance comparison of proposed approach with previous study.

Approach	Classifier	Class	Accuracy	PPV	Sensitivity	F1 score
Ensemble model^8^	SGR-VC	$\beta$-Thalassaemia non-carrier	0.93	0.96	0.93	0.93
		$\beta$-Thalassaemia carrier		0.89	0.89	0.90
		Weighted average		0.93	0.93	0.93
**Proposed approach**						
Scenario 3 (SMOTE)	RF	$\beta$-Thalassaemia non-carrier	0.96	0.96	0.95	0.96
		$\beta$-Thalassaemia carrier		0.95	0.96	0.96
		Weighted average		0.96	0.96	0.96
Scenario 3 (SMOTE)	ETC	$\beta$-Thalassaemia non-carrier	0.96	0.96	0.95	0.96
		$\beta$-Thalassaemia carrier		0.96	0.96	0.96
		Weighted average		0.96	0.96	0.96
Scenario 3 (ADASYN)	ETC	$\beta$-Thalassaemia non-carrier	0.96	1.00	0.92	0.96
		$\beta$-Thalassaemia carrier		0.93	1.00	0.96
		Weighted average		0.96	0.96	0.96

## Conclusion

The ratio of $\beta$-Thalassaemia carriers is increasing in Pakistan with a 5–7% current rate among the whole population which suggests that there is a dire need for an accurate and efficient approach for the detection of $\beta$-Thalassaemia carriers. This study proposes a machine learning-based approach for the classification of $\beta$-Thalassaemia carriers and $\beta$-Thalassaemia non-carriers to obtain high classification accuracy. In essence, two aspects are focused on: the dataset imbalance and the appropriate feature set. For data imbalance, SMOTE and ADASYN are analyzed for their efficacy to increase the accuracy and reduce models’ bias towards the major class. Keeping in view the fact that all features are not equally important, PCA and SVD are used to select important features which are unified to make a better feature set. Extensive experiments are performed involving different scenarios considering original data, oversampled data, and oversampled data with the unified framework of PCA and SVD features augmented with machine learning and deep learning models. Experimental results reveal that the proposed approach which integrates SMOTE with the unified framework of PCA and SVD yields the best results with 0.96 accuracy and surpasses the performance of existing approaches by 3.22%. Tree-based ensemble machine learning shows superior performance as compared to deep learning models. For future work, our goal is to increase the size of the dataset to improve the performance of deep learning models and achieve even better classification accuracy for $\beta$-Thalassemia carriers.

## Data Availability

The datasets generated and/or analysed during the current study are available on request. The dataset is not publicly available. The data can be requested from Furqan Rustam. All experimental protocols were approved by a Sheikh Zayed Hospital, Rahim Yar Khan, Pakistan.
